# Food Functional Factors and Nutritional Health

**DOI:** 10.3390/nu18152550

**Published:** 2026-08-04

**Authors:** Bowen Li, Guowei Le

**Affiliations:** 1 China-Hungary Belt and Road Joint Laboratory on Food Science, Chongqing Key Laboratory of Speciality Food Co-Built by Sichuan and Chongqing, College of Food Science, Southwest University, Chongqing 400715, China; 2 School of Food Science and Technology, Jiangnan University, Wuxi 214122, China

Diet fundamentally affects our long-term health. Over the past decade, the scientific understanding of this relationship has moved beyond simply counting calories or balancing macronutrients. Therefore, we recognize dietary habits as a combination of physiological needs, environmental influences, and conscious choices [1]. This shift requires us to look closer and deeper at how specific food components, particularly food functional factors, such as bioactive peptides, polyphenols, specific dietary fibers, and functional lipids, affect our metabolism. Recent research highlights how these specific nutritional inputs help maintain metabolic balance and influence cellular aging. More studies that explore these mechanisms, from molecular pathways to public health, aiming to turn nutritional science into practical, long-term health benefits.

The reality of the current food environment, which is increasingly dominated by ultra-processed foods (UPFs), should be addressed first before we get benefits from functional foods [2]. Even as our knowledge of nutrition grows, the convenience and high appeal of these processed products often put people toward unhealthy choices that override our natural fullness signals. These foods are typically loaded with altered nutrients, such as oxidized lipids and advanced glycation end products. Emerging evidence links them directly to the rising rates of obesity, type 2 diabetes, and cardiovascular diseases. For instance, a large-scale prospective cohort study recently published in The Lancet Regional Health—Europe clearly demonstrated that higher UPF intake is significantly associated with an increased risk of type 2 diabetes [3]. This risk goes beyond just high sugar and fat content. The breakdown of the natural physical structure of the food (the food matrix) and the pro-inflammatory nature of UPFs are also key drivers of metabolic harm. Common additives like emulsifiers can disrupt the gut microbiota, trigger intestinal inflammation and promote obesity [4]. In contrast, simply replacing a portion of UPFs in the diet with an equal amount of unprocessed or minimally processed foods can significantly lower the incidence of metabolic diseases. To protect public health moving forward, we need clearer policies and industry-wide food reformulation. Research should not only assess the harm of UPFs but also explore how integrating specific food functional factors into daily diets might offset some of these pro-inflammatory and metabolic disruptions.

In contrast to the risks associated with UPFs, intentional dietary patterns rich in functional factors offer profound therapeutic benefits. Increasing evidence demonstrates that whole-grain diets, caloric restriction, and specific amino acid limitations can improve insulin sensitivity, balance lipid profiles, and potentially extend health span. For instance, dietary methionine restriction (MR) has been shown to fundamentally alter lipid metabolism in ways that may promote longevity and ameliorate ulcerative colitis [5,6]. In preclinical models, MR reduced fat accumulation, enhanced insulin sensitivity, and reversed obesity by activating the FGF21 signaling pathway and downregulating hepatic lipogenic genes, such as Scd1. Interestingly, the transcription factor HNF4α has been identified as a key determinant of cellular sensitivity to MR, offering a promising target for precision nutrition to identify individuals who would benefit most from dietary MR intervention [7]. Of note, these targeted dietary approaches serve as powerful, non-pharmacological tools for preventing and managing chronic diseases. Moving forward, the field requires larger clinical trials to validate how various functional factors and targeted dietary approaches can serve as non-pharmacological tools for preventing and managing chronic diseases.

To fully understand how food functional factors lead to such broad health benefits, we should pay attention to the gut microbiota. Functional factors, such as prebiotics, dietary polyphenols, and complex carbohydrates, contribute to the development of the gut microbiome. These microbes metabolize functional factors to produce beneficial metabolites, which in turn regulate our energy metabolism, systemic inflammation, and gut barrier integrity. This makes the microbiome an excellent target for therapies utilizing probiotics, prebiotics, and emerging postbiotics to restore metabolic balance [8]. However, the field of microbiome-based nutrition needs to move past simply observing links and start proving cause and effect. We need to identify exactly which bacteria interact with specific functional factors to drive precise health outcomes, eventually bringing standardized, customized symbiotic treatments into regular clinical care [9].

Individuals metabolize food functional factors differently due to individual differences such as genetics and lifestyle. A one-size-fits-all approach to diet is no longer practical. While general, population-wide dietary guidelines provide a good baseline, they fail to meet the unique needs of groups like pregnant women, the elderly, athletes, or people managing conditions such as diabetes. This brings us to the necessity of personalized nutrition [10]. By analyzing an individual’s genetic makeup, metabolic profile, and microbiome, we can offer highly tailored advice on which functional factors will yield the greatest health benefits. Future research should focus on combining data from genomics and metabolomics to build reliable, algorithm-driven tools for personalized recommendations. Importantly, we need to translate these advanced scientific concepts into affordable and accessible strategies for everyday healthcare providers and the public.

Aligning with these advancements, our current issue presents a comprehensive synthesis of how bioactive components within complex food matrices modulate physiological health through intricate regulatory networks. Highlighting the intersection of aging and nutritional intervention, studies demonstrate that dietary supplementation with spinach-derived chlorophyll can significantly extend Drosophila lifespan and enhance stress resistance by remodeling detoxification networks via the CncC (Nrf2) signaling pathway [Contribution 1]. The collection further establishes edible bird’s nest (EBN) as a paradigm for multi-target regulation, where its unique protein-glycoprotein-sialic acid core and specific sialylated glycopeptides provide neuroprotection and alleviate inflammation across the gut–brain and gut-lung axes [Contributions 2–4]. Investigations into metabolic and organ health reveal that Coix seed oil effectively manages hyperuricemia by promoting uric acid homeostasis and intestinal integrity [Contribution 5], while camel milk proteins and lactopontin-fortified matrices are shown to mitigate colitis and support bone development through the modulation of the gut microbiota and the gut-bone axis [Contributions 6 and 7]. Additionally, the issue addresses technological and clinical translation, exploring advanced intestinal-targeted delivery systems for phenolic compounds to overcome bioavailability barriers [Contribution 8], as well as the efficacy of anti-inflammatory dietary education in improving the mental health and quality of life of breast cancer patients [Contribution 9]. These collective findings advance functional food research from a reductionist approach to a systems-biology perspective, providing critical academic support for precision nutrition and the development of evidence-based health interventions.

In conclusion, the relationship between food functional factors and nutritional health is profoundly complex and multifaceted, encompassing severe modern risks driven by UPFs as well as therapeutic opportunities offered by specific dietary patterns and microbiome modulation. The future of nutritional science clearly lies in a highly personalized, mechanism-based approach that fully harnesses the biological power of food functional factors. We encourage the scientific and medical communities to continue relentlessly exploring the fundamental regulatory principles of dietary habits on metabolic health. By promoting evidence-based diets rich in functional factors and integrating multi-omics technologies, we can work towards a future where precise, individualized nutrition serves as the ultimate cornerstone of preventive medicine.
